# Contrasting xylem vessel constraints on hydraulic conductivity between native and non-native woody understory species

**DOI:** 10.3389/fpls.2013.00486

**Published:** 2013-11-28

**Authors:** Maria S. Smith, Jason D. Fridley, Jingjing Yin, Taryn L. Bauerle

**Affiliations:** ^1^Department of Horticulture, Cornell UniversityIthaca, NY, USA; ^2^Department of Biology, Syracuse UniversitySyracuse, NY, USA

**Keywords:** hydraulic conductivity, xylem anatomy, embolism vulnerability, exotic woody plants, efficiency vs. safety, vessel connectivity

## Abstract

We examined the hydraulic properties of 82 native and non-native woody species common to forests of Eastern North America, including several congeneric groups, representing a range of anatomical wood types. We observed smaller conduit diameters with greater frequency in non-native species, corresponding to lower calculated potential vulnerability to cavitation index. Non-native species exhibited higher vessel-grouping in metaxylem compared with native species, however, solitary vessels were more prevalent in secondary xylem. Higher frequency of solitary vessels in secondary xylem was related to a lower potential vulnerability index. We found no relationship between anatomical characteristics of xylem, origin of species and hydraulic conductivity, indicating that non-native species did not exhibit advantageous hydraulic efficiency over native species. Our results confer anatomical advantages for non-native species under the potential for cavitation due to freezing, perhaps permitting extended growing seasons.

## Introduction

Wood xylem vessel members constitute the main pathway for water transport over long distances within a plant and are morphologically diverse across species. In addition to the pronounced differences in ring-porous vs. diffuse-porous species, xylem arrangement follows a continuum of organizational levels. Vessel organization and distribution including vessel number and frequency are associated with varying patterns of community assembly and adaptive variation in growth strategies across phylogentic groups (Baas, 1986). Moreover, correlations between wood anatomical traits (e.g., porosity type, variation in bordered pits and perforation types) and factors integrating seasonal water availability may reveal characteristics representing successful plant hydraulic properties such as water-use efficiency, conductivity, and vulnerability to cavitation (Jansen et al., 2004; Taneda and Sperry, 2008) that promote a physiological advantage of non-native over native species (Pratt and Black, 2006; Caplan and Yeakley, 2010).

High relative growth rate (RGR) is common to many introduced non-native woody plants (Dawson et al., 2011). Hydraulic architecture is associated with plant growth rate (Brodribb et al., 2002; Meinzer et al., 2010), with xylem vessel structure and size identified as the main constraints on maximum water transport and thus hydraulic conductivity (Tyree and Ewers, 1991; Steppe and Lemeur, 2007). Woodrum et al. (2003) examined xylem vessel anatomy and hydraulic conductivity of maples (*Acer*) of varying growth rates but few differences in hydraulic conductivity or anatomical xylem vessel characteristics were apparent. Pratt and Black (2006) were also unable to find differences in cavitation resistance or xylem specific conductivity between five pairs of co-occurring native and non-native trees. However, few if any studies on the relationship between vessel conduit anatomy and water transport in native vs. non-native species have been performed to date. Yet, trends in species' hydraulic conductivity have been broadly categorized by means of xylem conduit diameter and rate of water flow (efficiency; Tyree et al., 1994), and that there exists a positive relationship between vessel diameter (VD) and growth rate.

Plant water-use strategies are often evaluated as functional trade-offs that maximize resource capture and retention rates based on the resident environment (Grime, 2001). Non-native species with invasive potential are considered a threat to native plant communities due to more efficient resource-use (Grotkopp et al., 2002; Funk and Vitousek, 2007; Drenovsky et al., 2012) or higher resource use and capture (Cavaleri and Sack, 2010) such that they effectively out-compete natives in their introduced range (Davis et al., 2000; Drenovsky et al., 2012). One way in which non-natives may increase their efficiency is by decreasing the cost of acquiring or using resources. By investing in cheaper structural tissues (Dale and Causton, 1992), species lose benefits associated with increased construction cost which, in turn, influences species' hydraulic vulnerability to water stress or freezing (Carlquist, 1977). Elucidating the linkage between wood characteristics and water transport provides insight into plant hydraulic functional strategies (Baas, 1986; Taneda and Sperry, 2008).

Embolism vulnerability places a constraint on maximum water transport through a reduction in hydraulic conductance as a consequence of drought and freeze-thaw cycles (Pockman and Sperry, 1996; Meinzer et al., 2001) and even normal growth conditions (Cochard and Tyree, 1990; Sperry et al., 1994; McCully et al., 1998). Recent studies have emphasized additional vessel characteristics that are potentially important drivers of hydraulic transport and protection against embolism formation, including inter-vessel pit structure and size as a bottleneck to air-seeding (Jansen et al., 2004; Choat et al., 2008; Christman et al., 2009; Lens et al., 2011), vessel perforation plate type (openings at the end of vessel elements; Jansen et al., 2004), vessel wall thickness (Hacke et al., 2001), and vessel connectivity (Loepfe et al., 2007; Lens et al., 2011). Comparative studies that investigate vessel characteristics over a wide survey of plant species are limited (but see Jansen et al., 2004; McCulloh et al., 2010).

Here we examine the hydraulic properties of 82 native and non-native woody species common to forests of Eastern North America, including several congeneric groups, which represent a range of anatomical wood types. Broad surveys containing a large number of species have the potential to reveal functional classification syndromes that relate to plant productivity (Zanne et al., 2010). Our goal was to compare relationships between hydraulic conductance and theoretical xylem vessel vulnerability by stem vascular structure and arrangement between non-native and native woody species. We examined relationships between stem xylem vessel anatomical arrangement, xylem vessel types, stem specific hydraulic conductivity, wood development/ timing of vessel development, and cavitation vulnerability index among native and non-native woody understory species, as well as differences in water-use efficiency between native and non-native individuals. The use of an index to exam potential stem vulnerability is a useful metric when examining larger data sets that represent several species. Specifically, we tested the hypothesis that non-native understory species have greater capacity for water transport than native understory species.

## Materials and methods

### Plant material and growing conditions

Stem material was harvested from mature plants in a common garden comprised of a homogenous, clay loam soil in Syracuse, NY, USA (43°03′ N, 76°09′ W), representing a range of native and non-native woody species including several common genera (Table 1). At the time of harvest, individuals were approximately the same size and age and maintained under the same condition. Each species was grown in three replicate blocks, each under 80% shade cloth during the growing season (late May—late October) to simulate deciduous forest conditions. Species were obtained from natural areas in central New York when possible; those species not available in our region were sourced from commercial growers located in the northern US. Plants were pruned occasionally over the 5-year period preceding stem harvest but not fertilized or watered, and summer wilting was not observed.

**Table 1:** **Woody shrub species list tested and attributes**.

**Species**	**Family**	**Invasive status**	**Porosity type**	**Perforation plate type**	**VD (μm)**	**VF (N/mm2)**	**VI (VD/VF)**	**K*_s_*(10^−3^ kg s MPa^−1^ m^−3^)**
*Acer negundo*	Sapindaceae	Native	Diffuse	Simple	21.98	42.70	0.51	3.05
*Acer pensylvanicum*	Sapindaceae	Native	Diffuse	Simple	26.40	18.80	1.40	*
*Acer saccharum*	Sapindaceae	Native	Diffuse	Simple	20.35	44.90	0.45	*
*Berberis canadensis*	Berberidaceae	Native	Semi-ring	Simple	12.60	80.27	0.16	*
*Berberis koreana*	Berberidaceae	Non-native	Ring	Simple	16.77	62.90	0.27	8.69
*Berberis thunbergii v. atropurpurea*	Berberidaceae	Non-native	Semi-ring	Simple	12.37	113.70	0.11	3.23
*Berberis vulgaris*	Berberidaceae	Non-native	Ring	Simple	13.02	123.53	0.11	1.45
*Calycanthus floridus*	Calycanthaceae	Native	Semi-ring	Simple	26.00	35.70	0.73	*
*Celastrus orbiculatus*	Celastraceae	Non-native	Ring	Simple	22.11	32.10	0.69	4.52
*Celastrus scandens “diana”*	Celastraceae	Native	Ring	Simple	17.7	57.67	0.31	4.97
*Cephalanthus occidentalis*	Rubiaceae	Native	Ring	Simple	16.29	52.27	0.31	2.36
*Chionanthus virginicus*	Oleaceae	Native	Ring	Simple	20.6	24.40	0.84	3.96
*Cornus alternifolia*	Cornaceae	Native	Diffuse	Scalariform	26.43	31.20	0.85	1.12
*Cornus amomum*	Cornaceae	Native	Diffuse	Scalariform	28.49	30.80	0.92	3.02
*Cornus florida*	Cornaceae	Native	Diffuse	Scalariform	17.80	32.50	0.55	*
*Cornus mas*	Cornaceae	Non-native	Diffuse	Scalariform	15.91	28.60	0.56	5.45
*Cornus sericea*	Cornaceae	Native	Diffuse	Scalariform	23.6	70.60	0.33	6.23
*Diervilla lonicera*	Caprifoliaceae	Native	Diffuse	Scalariform	25.30	55.20	0.46	0.98
*Diervilla rivularis*	Caprifoliaceae	Native	Diffuse	Scalariform	22.60	42.40	0.53	*
*Dirca palustris*	Thymelaeaceae	Native	Diffuse	Simple	15.5	68.20	0.23	0.58
*Elaeagnus angustifolia*	Elaeagnaceae	Non-native	Diffuse	Simple	24.40	19.10	1.28	*
*Elaeagnus commutata*	Elaeagnaceae	Native	Semi-ring	Simple	16.50	62.36	0.26	*
*Elaeagnus multiflora*	Elaeagnaceae	Non-native	Ring	Simple	28.68	35.60	0.81	7.59
*Elaeagnus pungens*	Elaeagnaceae	Non-native	Diffuse	Simple	13.30	85.40	0.16	*
*Elaeagnus umbellata*	Elaeagnaceae	Non-native	Semi-ring	Simple	25.80	28.27	0.91	*
*Euonymus alatus*	Celastraceae	Non-native	Diffuse	Simple	15.86	56.00	0.28	1.29
*Euonymus americanus*	Celastraceae	Native	Ring	Simple	11.70	142.00	0.08	*
*Euonymus atropurpureus*	Celastraceae	Native	Diffuse	Simple	17.25	78.67	0.22	6.29
*Euonymus bungeanus*	Celastraceae	Non-native	Diffuse	Simple	18.18	72.40	0.25	10.1
*Euonymus europeaus “atrorubens”*	Celastraceae	Non-native	Semi-ring	Simple	15.70	91.47	0.17	*
*Euonymus hamiltonianus sieboldianus*	Celastraceae	Non-native	Diffuse	Simple	18.88	84.80	0.22	9.08
*Euonymus obovatus*	Celastraceae	Native	Semi-ring	Simple	10.60	157.58	0.07	*
*Euonymus phellomanus*	Celastraceae	Non-native	Diffuse	Simple	13.60	104.56	0.13	*
*Frangula alnus*	Rhamnaceae	Non-native	Semi-ring	Simple	20.8	31.30	0.66	0.25
*Frangula caroliniana*	Rhamnaceae	Native	Semi-ring	Simple	25.08	32.80	0.76	16.3
*Hamamelis virginiana*	Hamamelidaceae	Native	Diffuse	Scalariform	21.21	58.00	0.37	8.68
*Hydrangea arborescens*	Hydrangeaceae	Native	Semi-ring	Scalariform	21.55	51.10	0.42	1.22
*Hydrangea paniculata “Floribunda”*	Hydrangeaceae	Non-native	Diffuse	Scalariform	19.60	30.20	0.65	*
*Hydrangea quercifolia*	Hydrangeaceae	Native	Semi-ring	Scalariform	24.37	50.40	0.48	0.69
*Kolokowitzia amabilis*	Caprifoliaceae	Non-native	Ring	Scalariform	16.46	38.40	0.43	13.6
*Lindera benzoin*	Lauraceae	Native	Diffuse	Simple	16.21	36.90	0.44	0.24
*Lonicera canadensis*	Caprifoliaceae	Native	Diffuse	Simple	13.20	83.53	0.16	*
*Lonicera fragrantissima*	Caprifoliaceae	Non-native	Semi-ring	Simple	13.81	59.95	0.23	0.66
*Lonicera hirsuta*	Caprifoliaceae	Native	Diffuse	Simple	17.80	87.60	0.20	*
*Lonicera involucrata var involucrate*	Caprifoliaceae	Native	Semi-ring	Simple	15.12	93.53	0.16	1.70
*Lonicera japonica “halliana”*	Caprifoliaceae	Non-native	Diffuse	Simple	24.70	27.60	0.89	*
*Lonicera morrowii*	Caprifoliaceae	Non-native	Semi-ring	Simple	15.04	51.80	0.29	0.63
*Lonicera nitida*	Caprifoliaceae	Non-native	Semi-ring	Simple	11.77	120.40	0.1	8.79
*Lonicera oblongifolia*	Caprifoliaceae	Native	Semi-ring	Simple	14.10	56.90	0.25	*
*Lonicera periclymenum “GS Thomas”*	Caprifoliaceae	Non-native	Diffuse	Simple	24.80	44.07	0.56	*
*Lonicera pileata*	Caprifoliaceae	Non-native	Semi-ring	Simple	12.30	116.00	0.11	*
*Lonicera ruprechtiana*	Caprifoliaceae	Non-native	Semi-ring	Simple	16.06	50.50	0.32	4.21
*Lonicera sempervirens*	Caprifoliaceae	Native	Semi-ring	Simple	24.92	36.00	0.69	2.47
*Lonicera standishii*	Caprifoliaceae	Non-native	Diffuse	Simple	12.30	80.57	0.15	*
*Lonicera tatarica*	Caprifoliaceae	Non-native	Semi-ring	Simple	15.57	74.87	0.21	3.52
*Lonicera villosa var villosa*	Caprifoliaceae	Native	Semi-ring	Simple	14.10	57.50	0.25	*
*Lonicera x bella*	Caprifoliaceae	Non-native	Diffuse	Simple	16.50	64.00	0.26	*
*Lonicera xylosteum*	Caprifoliaceae	Non-native	Semi-ring	Simple	15.46	69.95	0.22	1.40
*Ptelea trifoliate*	Rutaceae	Native	Ring	Simple	23.99	12.44	1.93	0.21
*Rhamnus alnifolia*	Rhamnaceae	Native	Semi-ring	Simple	18.90	37.80	0.50	*
*Rhamnus cathartica*	Rhamnaceae	Non-native	Semi-ring	Simple	14.40	58.80	0.24	*
*Rhamnus davurica*	Rhamnaceae	Non-native	Ring	Simple	14.23	84.20	0.17	11.6
*Ribes rubrum “cherry”*	Grossulariaceae	Non-native	Diffuse	Scalariform	16.2	60.00	0.27	1.56
*Rosa multiflora*	Rosaceae	Non-native	Diffuse	Simple	16.09	54.17	0.3	4.65
*Rosa palustris*	Rosaceae	Native	Semi-ring	Simple	19.90	48.20	0.41	*
*Sambucus nigra ssp canadensis*	Adoxaceae	Native	Diffuse	Simple	27.52	28.60	0.96	0.17
*Sambucus racemosa*	Adoxaceae	Native	Ring	Simple	26.45	42.00	0.63	3.46
*Shepherdia argentea*	Elaeagnaceae	Native	Ring	Simple	18.55	43.00	0.43	4.39
*Shepherdia canadensis*	Elaeagnaceae	Native	Diffuse	Simple	16.49	85.50	0.19	2.27
*Stephanandra incisa “crispa”*	Rosaceae	Non-native	Diffuse	Simple	16.50	34.40	0.48	*
*Viburnum acerifolium*	Adoxaceae	Native	Diffuse	Scalariform	14.50	68.80	0.21	*
*Viburnum dentatum*	Adoxaceae	Native	Diffuse	Scalariform	22.4	38.80	0.58	4.45
*Viburnum dilatatum*	Adoxaceae	Non-native	Diffuse	Scalariform	20.06	51.53	0.39	2.32
*Viburnum edule*	Adoxaceae	Native	Diffuse	Scalariform	27.22	50.20	0.54	3.93
*Viburnum lantana*	Adoxaceae	Non-native	Diffuse	Scalariform	20.54	58.10	0.35	1.16
*Viburnum lentago*	Adoxaceae	Native	Diffuse	Scalariform	22.06	51.00	0.43	2.70
*Viburnum nudum ssp cassanoides*	Adoxaceae	Native	Semi-ring	Scalariform	15.90	65.80	0.24	*
*Viburnum opulus*	Adoxaceae	Non-native	Diffuse	Scalariform	22.10	63.50	0.35	*
*Viburnum opulus var Americana*	Adoxaceae	Native	Diffuse	Scalariform	19.26	71.50	0.27	4.21
*Viburnum prunifolium*	Adoxaceae	Native	Diffuse	Scalariform	15.00	46.70	0.32	*
*Viburnum rafanesquianum*	Adoxaceae	Native	Diffuse	Scalariform	16.76	66.60	0.25	26.3
*Viburnum setigerum*	Adoxaceae	Non-native	Diffuse	Scalariform	20.20	45.60	0.44	*

Mean vessel traits, VD, vessel diameter; VF, vessel frequency; VI, vulnerability index; and Ks, stem hydraulic conductivity, for each species.

* Denotes species without high pressure flow meter (HPFM) data.

### Hydraulic conductivity and WUE measurements

Three terminal branch stems of similar diameter containing 1 year's growth were randomly sampled from individuals of each species from each of three replicate blocks in November 2011. Stems were kept moist in damp paper towels in a cooler ~2 h prior to taking measurements in a temperature-controlled room at 25°C, equal to the temperature of the high pressure flow meter (HPFM) (Dynamax Inc., Houston, TX, USA). Diameter and stem length were recorded, and the cortex was shaved from the proximal end of stems prior to attachment to a HPFM.

Stem hydraulic conductance (*K*_*h*_) was measured directly with the HPFM, using methods described by Tyree et al. (1995). Each measurement was recorded ~30 s after stems had a visible flow of water through the end of the stem. Conductance (*K*_*h*_, kg s MPa^−1^), the inverse of resistance, was measured by the force of pressurized water through the stem (*P*) (MPa m^−1^) and the rate of water flow (*F*) (Kg s^−1^). Conductance was calculated as the slope of the regression plot *F* vs. *P*:

(1) $$K_{h}=dF/dP$$

Stem hydraulic measurements were conducted using quasi-steady state, where *F* and *P* are approximately constant (Tyree et al., 1993, 1995). Specific stem hydraulic conductivity (K*_s_*) was calculated factoring out the variation in stem length and diameter cross-sectional area (kg s MPa^−1^ mm^−3^, Sperry et al., 1988).

Photosynthesis was monitored monthly for each individual at intensities of 800, and 100 mmol photon^−2^ s^−1^, 700 mmol s^−1^flow rate, 20°C (Fridley, 2012). WUE was calculated as the ratio of carbon fixed to water lost, (Li-COR 6400, Inc., Lincoln, NE, USA) (C uptake/transpiration rate, in units of micromol CO2 per mmol H2O).

### Anatomical measurements

Two to three stem segments used for hydraulic conductivity were used for anatomical sectioning following conductivity measurements. In addition, three supplemental stem segments collected from the same plants in November 2010 were also sectioned for anatomical measurements. One cm long segments in random locations were removed from the stem, immediately preserved in formalin-acetic acid-alcohol solution (FAA), and stored at room temperature until the embedding process. Samples were dehydrated in a series of ethanol-tertiary butanol (TBA) dilutions before infiltration with pure TBA (Ruzin, 1999).

Stem anatomical samples were embedded in successive changes of Paraplast Plus embedding medium (McCormick Scientific, Saint Louis, Missouri, USA) in a 60°C drying oven for 2 days. Samples were embedded in a final paraffin change hardened with 15% (v:v) paramount (Fischer Scientific, Fair Lawn, New Jersey, USA). Transverse cross-sections were cut at 20 μm increments using a rotary microtome (HM 355S, Microm International GmbH, Walldorf, Germany). Cross sections were stained with saffranin-O [1% (w:v) in 50% ethanol] and counter-stained using fast-green [0.1% (w:v) in 1:1 absolute ethanol and clove oil] in a series with histo-clear (National Diagnostics, Atlanta, Georgia, USA) to remove paraffin.

Five images per stem were randomly selected for imaging using 20x magnification with a compound light microscope with a fixed camera attachment (Olympus Imaging Corp., Tokyo, Japan). Images representing 0.77 mm^2^ cross sectional area were first processed through Photoshop (CS5; Adobe Systems Inc., Mountain View, CA, USA) to select and fill each individual vessel, and then analyzed for xylem vessel lumen cross sectional area (VA) using the image-analysis software Image J (National Institute of Health, Bethesda, MD, USA, http://rsb.info.nih.gov/ij/index.html). Vessel area was converted to diameter (VD) assuming circularity of vessels. Conduit-containing sections of each image were then randomly cropped to 0.09 mm^2^ to represent the xylem area of the smallest stem to obtain vessel frequency (VF) over a uniform area for all species. The use of an index can be a valuable metric to examine larger data sets that are compiled of several species to look for generality in vulnerability patterns (Zanne et al., 2010). The potential of vessel vulnerability during water stress was determined using methods from Carlquist (1977) where vulnerability index (VI) = VD/VF, where VD is vessel diameter (μm), and VF is VF (N/mm^−2^) (Gonçalves et al., 2007; Bauerle et al., 2011; Aref et al., 2013). To parse seasonal differences in vessel traits and vulnerability, three equal concentric rings were overlaid on the cross section to delineate the first, middle, and last rows of vessels to represent the seasonal transition in vessel development from early to late season. Vessel measurements within the three rings were analyzed using the same method as above. Xylem vessel diameter of all 82 species was classified into seven classes from <10 μm to >35 μm at 5 μm intervals. The frequency of each class was estimated.

Vessel groupings were classified into four categories in both metaxylem, the primary xylem that differentiates after the protoxylem and is characterized by broader vessels, and secondary xylem, i.e., the categories of 2, 3, 4, and more than 5 vessels grouped together. The amount of vessel groupings in each classification was counted. For each classification we then calculated grouped vessel percentage (the percentage of the number of vessels grouped relative to the total number of vessels), vessel grouping index (mean number of vessels per vessel grouping), and the percentage of solitary vessels relative to the total number of vessels.

### Statistical methods

Differences in the distribution of vessel class frequency were assessed between native and non-native understory species using the Chi-square test. Differences in factors predicting conductivity, vessel traits and vulnerability index were tested using mixed-effect models to control for variability from genus classification. A bivariate regression analysis tested for significance in relationships between conductivity and vessel traits, where conductivity and vulnerability index were log-transformed to improve the assumption of normality. A linear 90th quantile regression was performed using the “quantreg” package from R (v. 2.13.1) to estimate the slope of the “packing limit” of vessels, representing the upper limit of the number of vessels that can fit in a given area based on size. Forty-nine of the 82 species, those with conductivity measurements, were analyzed for significance in VD over three distinct rows of xylem vessels, representing three different periods of annual wood formation (the first, middle and last row of selected vessels). A linear mixed model was constructed to determine how time influences vessel sizes between porosity type, origin, and perforation plate type. Genus was included as a random variable, and vessel row was used as an interaction term for time across effects. Vessel grouping in metaxylem was only compared between non-native and native species that had metaxylem using the Mann-Whitney *U*-test. Each classification of vessel grouping in secondary xylem was compared, respectively between non-native and native species using One-Way analysis of variance (ANOVA). Data were tested for normality and homogeneity to determine if it matched the assumptions of ANOVA. A mixed effects model was used to test for significant predictors of water use efficiency (WUE), using origin and differing light levels across 38 of the 82 species. Species was used as random factor, due to non-independence of repeated measures. Tukey's HSD *post-hoc* analysis was performed to distinguish differences between light levels. The relationships between vulnerability index and vessel groupings traits were examined individually by linear regression. All analyses excluding quantile regression were performed using JMP (SAS Institute Inc., Cary, NC, v. 10.0).

## Results

Of the 82 species studied, porosity type did not differ with origin (*P* = 0.970). When species were separated by porosity type, 55.3% of native species had a diffuse porous xylem ring structure, 29.8% had a semi-ring, and 14.9% had a ring porous vessel. Non-native species had a distribution comprised of 52.6% diffuse porous, 31.6% semi-ring porous, and 15.8% ring porous. However, contributions of some overrepresented genera drove much of the porosity type distribution. When accounting for genus, members of *Viburnum* and *Lonicera* comprised ~37% of the total individuals, with most species within a genus sharing similar perforation plate type and porosity type traits (Table 1).

### Vessel size distribution

**Figure 1:**
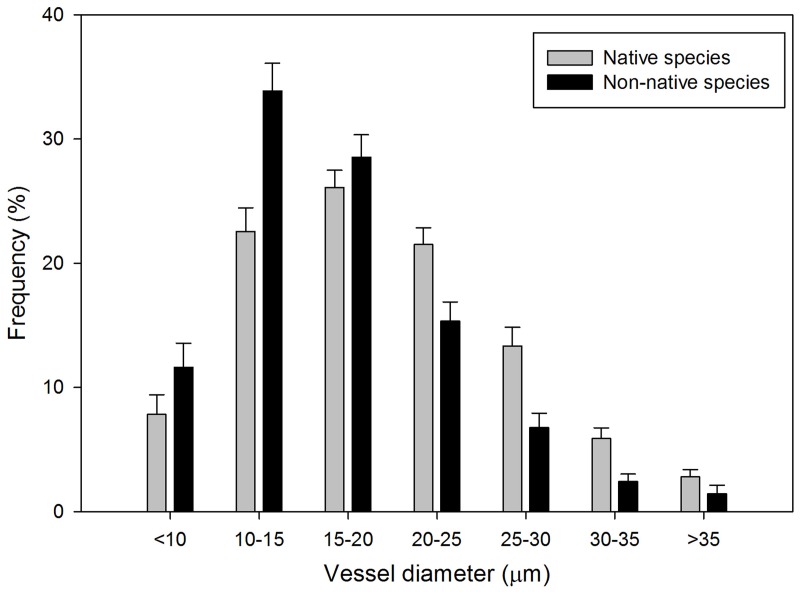
**Frequency distribution of xylem vessel diameter (μm) (± 1 *SE*) for 52 native (gray bars) and non-native (black bars) woody shrub species**.

The distribution of vessel class frequency differed between native and non-native species (*P* < 0.0001, Figure 1). Vessels with diameters less than 20 μm appeared more frequently in non-native species than native species. The diameter of more than 50% of the vessels in non-native species ranged from 10 to 20 μm, in which 33.9% of the vessels had diameters between 10 and 15 μm. Frequency of the vessels with diameters more than 20 μm was higher in native species than non-native species. The diameter of more than 70% of the vessels in native species ranged uniformly from 10 to 25 μm, with the 15–20 μm interval class having the highest VF, 26.1%.

### Hydraulic conductivity (K*_s_*)

The mixed-effects model did not show any effects from porosity, perforation plate or status as native or non-native on hydraulic conductivity (*P* > 0.10).

### Vessel traits and vulnerability index

No differences in VD, VF, or VI were found across porosity type, origin or perforation plate type (*P* > 0.10, Table 3). Despite an insignificant difference, non-native species had lower log VI (−0.978 ± 0.167) compared with native species (−0.880 ± 0.141), which was the result of a lower but significant VD of non-native species at α = 0.10 (*P* = 0.0694). Overall, non-native species had smaller vessels and an overall lower vulnerability index.

### Relationships between VD, VF, VI, vessel area and K*_s_*

**Figure 2:**
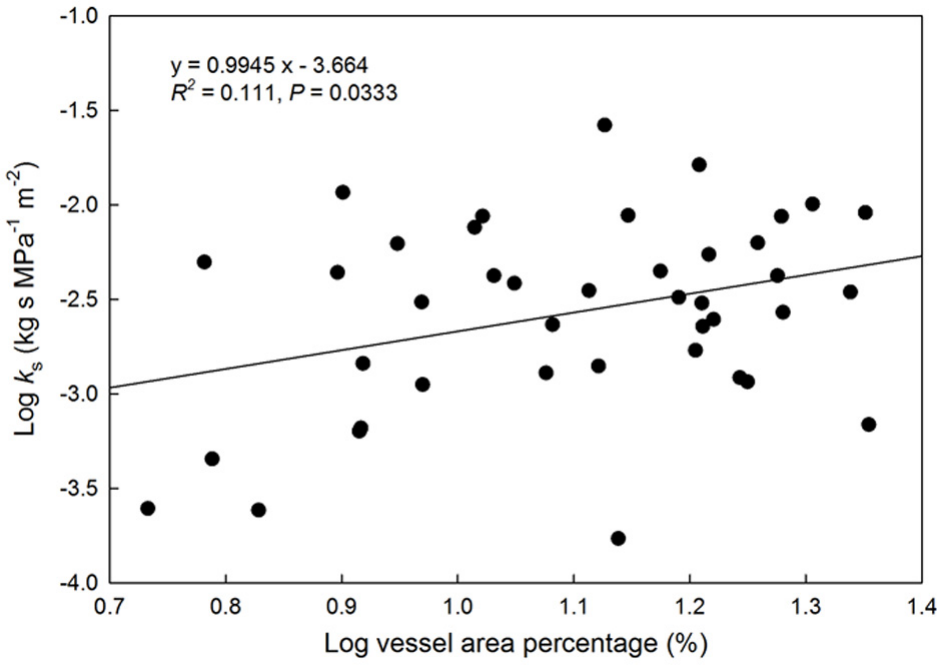
**Relationship between log(vessel area) as a percentage of the total viewing area vs. hydraulic conductivity (kg s MPa^−1^ m^−2^)**. Solid line represents the line of best fit (log[K*_s_*] = 0.095(log[vessel area %]) + 3.66). Points represent species with available HPFM data.

**Figure 3:**
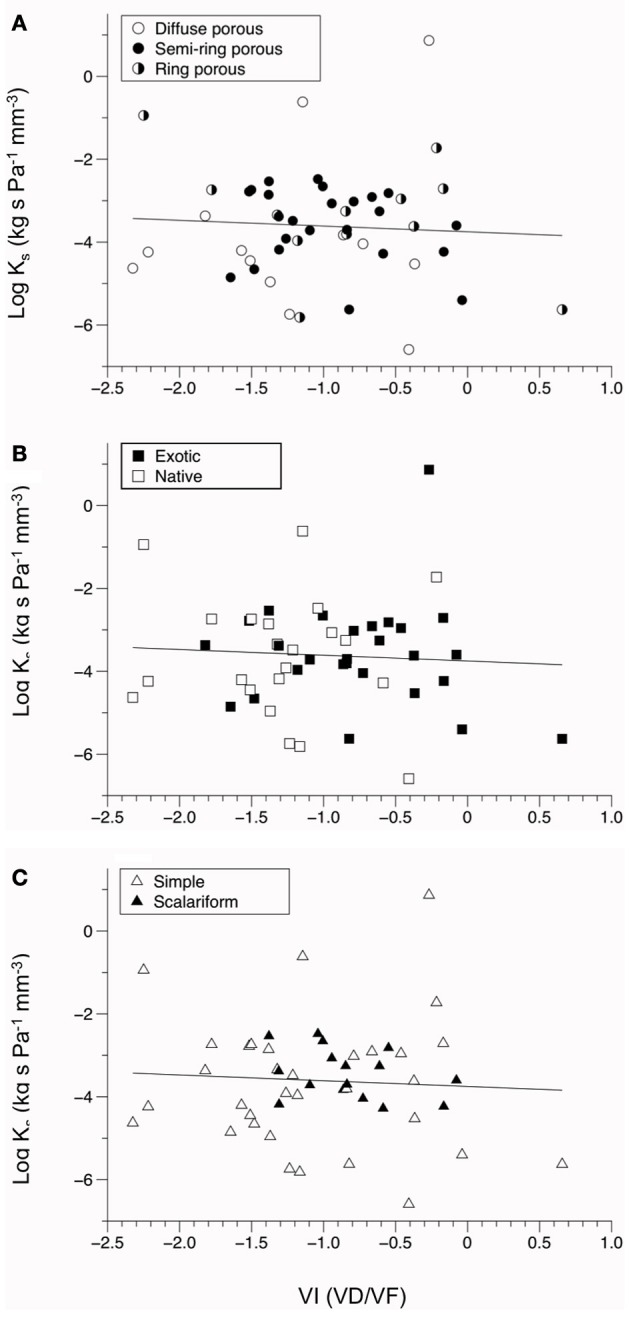
**Bivariate fit of vulnerability index (VI) vs. the log values of stem conductivity (K*_s_*). (A–C)** show VI by porosity, **(A)** by origin **(B)**, and by perforation plate type **(C)**. All relationships with log(K*_s_*) were not significantly correlated (*P* > 0.10).

A positive relationship was observed between log-transformed vessel area and log-transformed hydraulic conductivity (*P* = 0.033, Figure 2). This indicates faster water movement in species with a higher number of vessels per xylem area than those with less vessel area per unit xylem area (Figure 3). There was no correlation between K*_s_* and VI (*P* = 0.6677, Figure 3). When VI was divided into the individual components of VD and VF to test for a relationship with *K*_*s*_, no relationship was found for either VD (*P* = 0.3565) or VF (*P* = 0.380).

### Vessel packing

**Figure 4:**
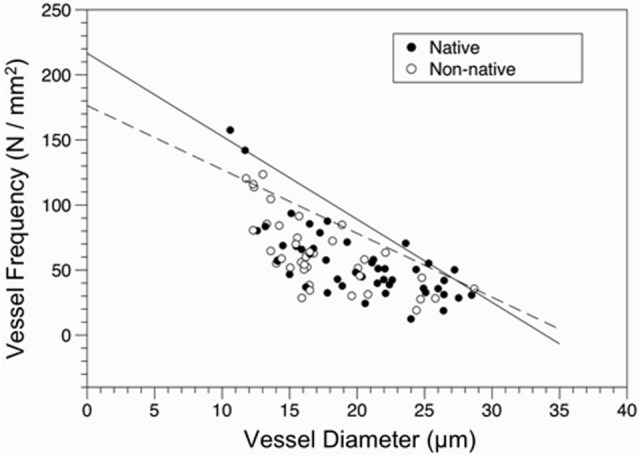
**Constraint relationship of vessel frequency (VF, N/mm^2^) vs. diameter (VD, μm) for native and non-native species**. Solid line represents the fit of the 90th quantile of native species (VF = 216.674-6.382 × VD), the dashed line for the 90th quantile of non-native species (VF = 176.386-4.909 × VD). Points are shown for origin (Closed circles = native, open circles = non-native).

Overall, an inverse relationship between VD and VF was found for both native and non-native species (Figure 4), indicating the larger the conduit diameter, the fewer number of conduits that can occupy a given area of wood. The slope of the constraint line for native species (−6.382) was steeper than the slope for non-native species (−4.909).

### Timing of formation and influence on vessel size

Genera explained 39% of the total variability within the model (*P* < 0.10), suggesting that variation among genera contributes to differences in VD. Timing of vessel development was a significant factor (*P* < 0.001). In a comparison of vessel rows, the last row of vessels was significantly smaller than that of both the first and middle rows (*P* < 0.10, Table 2). No interaction was detected between timing of vessel development and porosity, perforation plate type, or origin (*P* > 0.10). Only origin had an effect on VD (*P* < 0.001). Porosity type and perforation plate type had no predictive effect on VD (Table 3). The variability contributed by vessel row was 16%, compared with 32% for genera. Again, origin was the only significant factor in predicting VD (*P* < 0.0001).

**Table 2:** **The effect of vessel development timing, perforation plate type, porosity, origin, and interactions on vessel diameter (VD, μm)**.

**Factor**	**VD (μm)**	***F***	***P***
Vessel row		11.664	**<0.0001**
First	20.003 (0.904)		
Middle	20.889 (0.904)		
Last	17.215 (0.904)		
Perforation plate type		1.092	0.309
Porosity		1.035	0.364
Perf plate x vessel row		2.065	0.132
Porosity x vessel row		0.648	0.630
Origin		25.823	**<0.0001**
Native	21.234 (0.839)		
Non-native	17.505 (0.882)		
Origin × vessel row		0.089	0.915

Significant effects are in bold.

**Table 3:** **Vessel grouping in metaxylem and secondary xylem of native and non-native species**.

	**Native**	**Non-native**	***P-value***
**VESSEL GROUPINGS IN METAXYLEM**			
Number of 2-vessel groupings	4.51 (0.60)	4.84 (0.73)	*n.s*.
Number of 3-vessel groupings	3.10 (0.48)	3.06 (0.42)	*n.s*.
Number of 4-vessel groupings	1.71 (0.61)	2.16 (0.29)	*n.s*.
Number of >5-vessel groupings	1.96 (0.58)	4.27 (0.82)	**0.0184**
2-vessel grouping (%)	21.27 (2.94)	19.02 (2.94)	*n.s*.
3-vessel grouping (%)	16.92 (1.36)	12.08 (1.94)	*n.s*.
4-vessel groping (%)	9.83 (1.28)	7.23 (0.73)	*n.s*.
>5-vessel grouping (%)	9.69 (2.12)	12.08 (1.22)	*n.s*.
Vessel grouping index	3.14 (0.13)	3.44 (0.14)	*n.s*.
Solitary vessels (%)	13.97 (4.11)	22.80 (3.86)	*n.s*.
**VESSEL GROUPINGS IN SECONDARY XYLEM**			
Number of 2-vessel groupings	21.51 (2.07)	17.46 (2.08)	*n.s*.
Number of 3-vessel groupings	4.99 (0.45)	3.29 (0.42)	**0.0114**
Number of 4-vessel groupings	3.30 (0.35)	1.29 (0.23)	**<0.0001**
Number of >5-vessel groupings	3.00 (0.44)	2.65 (0.85)	*n.s*.
2-vessel grouping (%)	23.72 (1.72)	19.70 (1.92)	*n.s*.
3-vessel grouping (%)	9.04 (0.87)	5.86 (0.77)	**0.0099**
4-vessel groping (%)	7.46 (0.82)	2.77 (0.43)	**<0.0001**
>5-vessel grouping (%)	9.60 (2.02)	5.57 (1.09)	*n.s*.
Vessel grouping index	2.61 (0.06)	2.43 (0.07)	**0.0271**
Total solitary vessels (%)	51.43 (2.98)	60.49 (2.52)	**0.0277**

Significant P-values for the comparisons are in bold; n.s. indicates a non-significant difference.

### Vessel groupings

Approximately 62% of non-native species had metaxylem, while only 16% of native species had metaxylem. Comparing the vessel groupings in metaxylem of the non-native species to the few native species that had metaxylem, non-natives had a higher number of vessel groupings in the ≥5-vessel grouping class (*P* = 0.0184, Table 3).

In the secondary xylem, native species had significantly more vessel groupings in the 3- (*P* = 0.0114) and 4-vessel grouping classes (*P* < 0.0001). The proportion of vessel groupings to total vessels was also significantly higher in native species, compared with non-natives, in these two categories (*P* = 0.0099 for the 3-vessel grouping class and *P* < 0.0001 for the 4-vessel grouping class). Thus, the vessel grouping index in native species was higher than that in non-native species (*P* = 0.0271); in other words, non-native species had a higher proportion of solitary vessels in the secondary xylem than native species (*P* = 0.0277).

**Figure 5:**
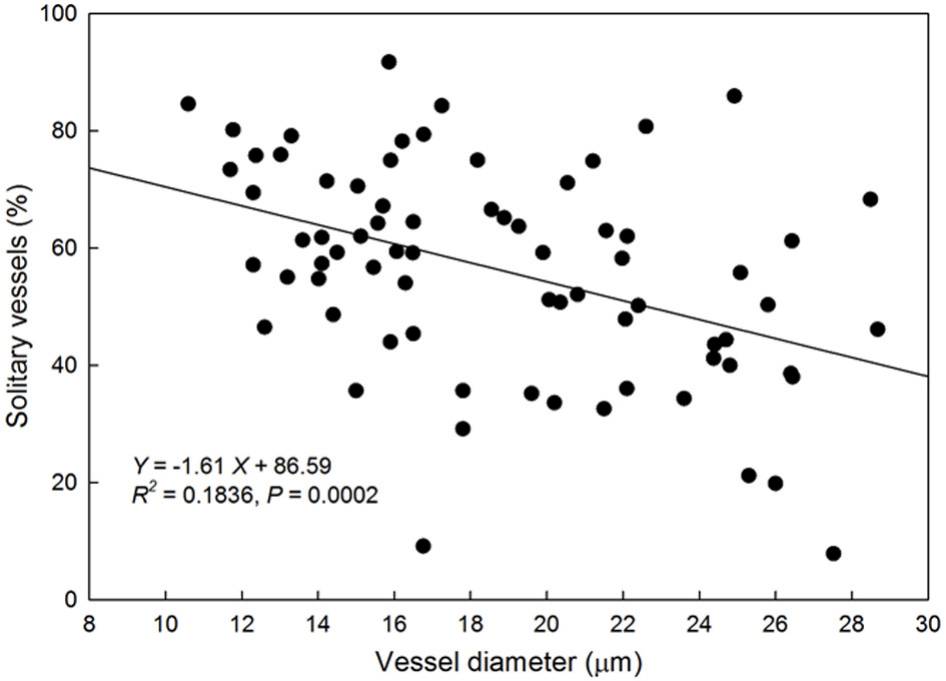
**Relationship between vessel diameter (μm) and the proportion of solitary vessels to total vessels in the xylem cross-section of each species**. Solid line represents the line of best fit (percentage of solitary vessels) = −1.61 (vessel diameter) + 86.59).

There was a negative linear relationship between average VD and proportion of solitary vessels to total xylem vessels (Figure 5) suggesting that species with smaller vessels tend to have fewer vessel groupings.

**Figure 6:**
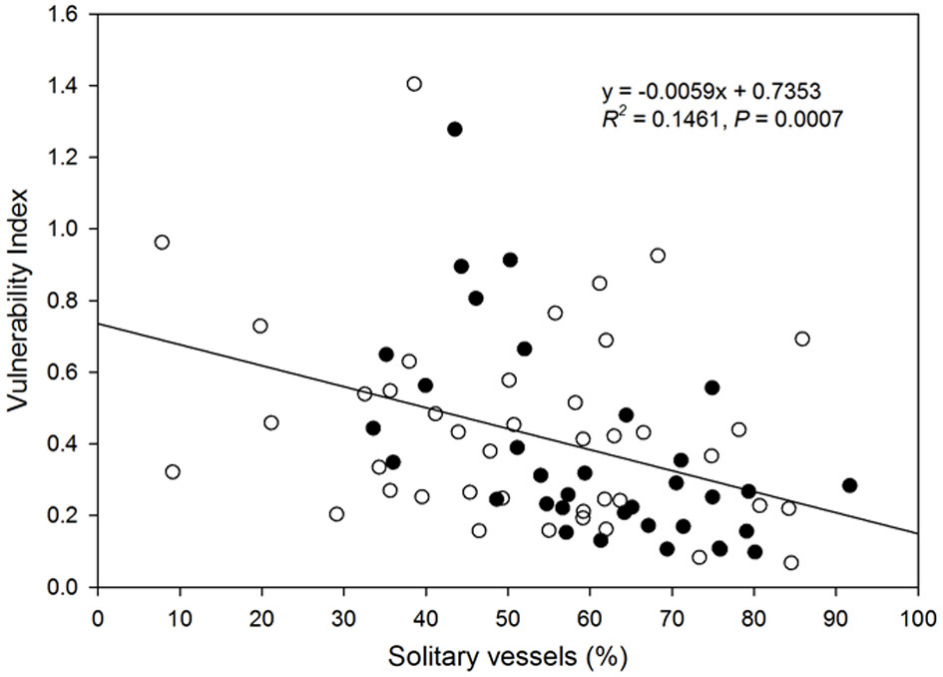
**Relationship between vulnerability index and proportion of solitary vessels to total vessels in the xylem cross-section of each species**. Filled circles represent native species, and open circles represent native species. Solid line represents the line of best fit (vulnerability index) = 0.0059 (solitary vessel) + 0.7353).

**Figure 7:**
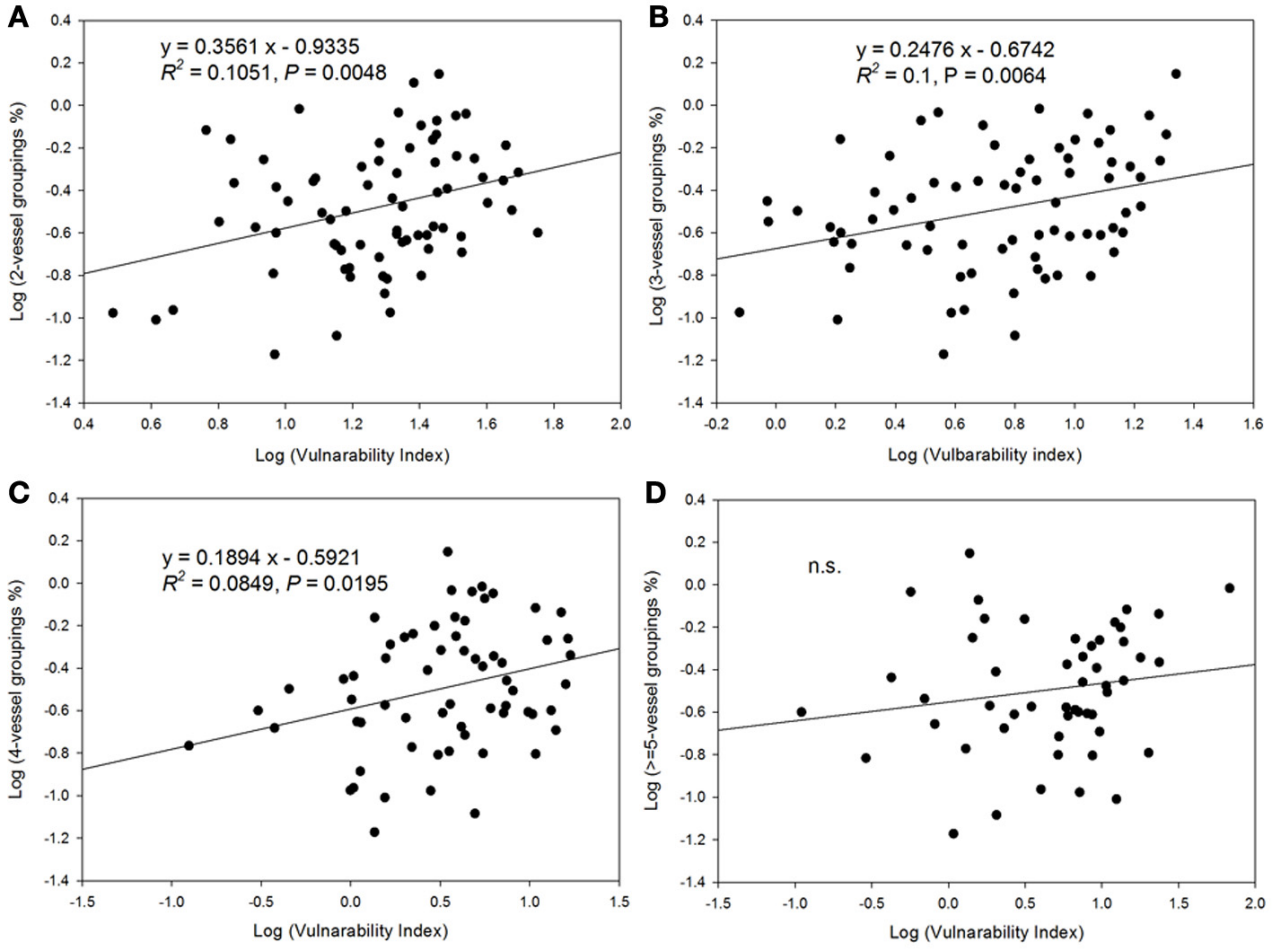
**Relationship between vulnerability index and vessel grouping classifications. (A)** the relationship with 2-vessel groupings; **(B)** 3-vessel groupings; **(C)** 4-vessel groupings; and **(D)** ≥5-vessel groupings.

There was a decrease in the vulnerability index with increasing percentage of solitary vessels (Figure 6). Specifically, the vulnerability index decreased with decreasing percentage of vessel groupings in the 2, 3, and 4-vessel grouping classes, but not in ≥5-vessel grouping class (Figure 7).

### Water use efficiency

Species represented 68.1% of the total variability in the model relative to the fixed factors, significantly contributing to differences in WUE (*P* < 0.0001). Light level was the only significant predictor of WUE, increasing 0.3 units per increase in light level (Table 4, *P* < 0.0001). Origin had no effect on WUE. All four light levels were significantly different from each other; WUE increased as light levels increased (*P* < 0.10).

**Table 4:** **The effect of porosity type, native status, perforation plate type, and light level on water use efficiency (WUE)**.

**Factor**	**WUE**	***SE***	***F***	***P***
**NATIVE STATUS**				
Native	2.75	0.12	0.3973	0.3973
Non-native	2.89	0.11		
**LIGHT LEVEL (PAR μ mol m^−2^ s^−1^)**				
50	1.38^a^	0.095	523.5904	**<0.0001**
100	2.27^b^	0.095		
300	3.58^c^	0.095		
800	4.06^d^	0.095		

Significant factors are in bold. Different letters in a column indicate significant differences in WUE between light levels at the P < 0.05 level.

## Discussion

Hydraulic contrasts in vessels between native and non-native species have been proposed in a number of recent studies (Pratt and Black, 2006; Caplan and Yeakley, 2010; Cavaleri and Sack, 2010). However, we present results from the first study to examine the direct relationship between xylem vessel anatomical characteristics and water flow across such a large diversity of native and non-native woody shrub species. Contrary to our hypothesis, we found non-native woody species possessed smaller secondary xylem vessels compared with native species although the two groups had similar hydraulic conductance (Figures 1, 3). Non-native, fast-growing species are often considered hydraulically efficient in that they exhibit xylem vessel characteristics that confer high water transport and reduced cavitation resistance (Gilbert et al., 2006; Markesteijn et al., 2011). The Hagen-Poiseuille law, which predicts that the hydraulic efficiency of a vessel increases with the fourth power of its diameter (Tyree and Zimmermann, 2002), would suggest that fast-growing non-native species should have wider VD conduits, which would be advantageous for a rapid growth strategy.

No significant differences in WUE efficiency were found between native and non-native species (Table 4, *P* = 0.3973). WUE has been postulated to be an important factor contributing to success of non-natives (Funk and Vitousek, 2007), yet differences in WUE between non-native and native species is contingent upon growth form and climate (Cavaleri and Sack, 2010). In co-occurring species of the same growth form, there is evidence to suggest that native and non-native species do not differ in WUE, since WUE may act in conjunction with variations in other plant traits to weaken or remove competitive advantages (Daehler, 2003; Funk and Vitousek, 2007; McAlpine et al., 2008; Cavaleri and Sack, 2010).

We found a significant positive correlation between xylem area as a percentage of wood area and hydraulic conductivity, which is consistent with Gleason et al. (2012) who found a positive correlation between xylem area and stem hydraulic conductivity across 120 Australian woody dicot species. However, there was not a relationship between conductivity and conduit traits of diameter and frequency, potential vulnerability index, and categorical porosity type (Figure 2). Since potential vulnerability index was calculated directly from VD, it is unlikely that this measure would relate to K*_s_* given the lack of relationship with VD. There was also no difference in conductivity between simple and scalariform plate types (*P* > 0.10), despite evidence from Christman and Sperry (2010) showing a considerable influence of vessel perforation plate type on xylem flow resistance in scalariform species. This finding may be due to the significant relatedness between individuals, since perforation plate type is conserved within groups of closely related species (Table 1). The relationship between hydraulic conductivity and resistance to cavitation events is well studied, with numerous findings of a trade-off between safety and efficiency (Pockman and Sperry, 2000; Hacke et al., 2006; Sperry et al., 2008; Markesteijn et al., 2011). The extent of this relationship is highly dependent upon adaptation of the xylem vessels (Markesteijn et al., 2011). A lack of difference in hydraulic conductance and potential vulnerability index in our study suggests that non-native plants do not exhibit a hydraulic advantage over native plants, a similar finding to that of Pratt and Black (2006). However, our lack of difference in hydraulic conductance and potential vulnerability index may be attributed to other anatomical factors such as perforation pits, which may account for >50% of total flow resistance of vessel networks depending on number, size, and structure (Wheeler et al., 2005; Choat et al., 2008; Lens et al., 2011) and conduit length which is correlated with porosity type and conductivity per xylem cross-sectional area (K_*XA*_; Zimmermann and Jeje, 1981; Lens et al., 2011). Additionally, our lack of difference may be due to the phylogenetic relationships among the species tested. Recent findings from Markesteijn et al. (2011) indicate that species differences can account for 62–98% of the variation in vessel traits. In our study, phylogenetic relatedness accounted for 39–68% of the total variation among traits. Comparing phylogenetic relatedness of native and non-native plants provided more meaningful explanation of invasive traits (McDowell, 2002; Dawson et al., 2011). However, phylogenetic relatedness may constrain morphological and physiological variations between species (Goldberg, 1987), which may explain the lack of difference in vessel traits observed in our study.

Non-native species had much higher (≥5) vessel groupings in metaxylem than native species. Metaxylem most likely becomes non-functioning after secondary xylem has developed, but it serves an important function during initial growth as in our species with only 1 year of growth. It is likely that the formation of metaxylem and vessel connectivity play an important role in the high growth rate of many non-native species. Maximum hydraulic conductivity has been found to increase with vessel connectivity (Loepfe et al., 2007). Meanwhile, higher vessel groupings may also increase the vulnerability to cavitation by increasing the probability for the spread of embolism (Loepfe et al., 2007). In contrast to Loepfe et al. (2007), Carlquist (2009), and Lens et al. (2011) stated that vessel grouping would decrease the vulnerability to cavitation since it serves to bypass frequent embolisms by providing alternative routes for water flow. In our study, higher vessel groupings, especially the 2-, 3-, and 4-vessel groupings, showed increasing vulnerability to embolism, supporting the Loepfe et al. (2007) model for the potential of vessel connectivity to promote embolism. Interestingly, non-native species also had a higher proportion of small solitary vessels within their secondary xylem, which could partially explain the lack of difference in K*_s_* between native and non-native species.

Average xylem vessel size was significantly smaller in non-native woody shrub species than in native species. In northern temperate deciduous forests, resistance to cavitation is an important feature in freeze tolerance. Davis et al. (1999) suggested a strong correlation between VD and cavitation by freezing, where small-vessel conduits are relatively resistant to cavitation. Thus, the smaller VD in non-native species may increase the competitive advantage of non-native species by allowing a longer growing season through higher resistance to cavitation from late-season freeze events. In fact, recent work by Fridley (2012) has shown that non-native deciduous plants retain leaves longer through the autumn season than related native species thus allowing prolonged growth.

While our study incorporates abroad range of species, our scope is limited to stem hydraulic conductance, which expressed inversely as resistance might account for a fraction of the total hydraulic resistance of a plant from roots to leaves (Tyree and Ewers, 1991; Becker et al., 1999). Root mean VDs were on average 30% larger than twig vessels in a recent study of tropical trees by Schuldt et al. (2013), indicating an 85% increase in theoretical hydraulic conductance, as calculated by the Hagen-Poiseuille law. In future work, having a complete hydraulic architecture of non-native plants may better elucidate competitive mechanisms for water transport in non-native species. Moreover, a thorough evaluation of hydraulic conductance throughout an entire growing season is warranted as we suspect that larger differences in K*_s_* may occur earlier in the spring when non-native species have functional metaxylem during shoot elongation.

## Author contributions

Taryn L. Bauerle and Jason D. Fridley designed the experiment. Maria S. Smith carried out the measurements. Maria S. Smith and Jingjing Yin performed the data analysis. Maria S. Smith, Taryn L. Bauerle, Jingjing Yin, and Jason D. Fridley prepared the manuscript.

### Conflict of interest statement

The authors declare that the research was conducted in the absence of any commercial or financial relationships that could be construed as a potential conflict of interest.
